# A review on shellfish polysaccharides: Extraction, characterization and amelioration of metabolic syndrome

**DOI:** 10.3389/fnut.2022.974860

**Published:** 2022-09-13

**Authors:** Xingwei Xiang, Qihong Jiang, Hongshun Yang, Xuxia Zhou, Yufeng Chen, Hui Chen, Shulai Liu, Lin Chen

**Affiliations:** ^1^College of Food Science and Technology, Zhejiang University of Technology, Hangzhou, China; ^2^Key Laboratory of Marine Fishery Resources Exploitment and Utilization of Zhejiang Province, Hangzhou, China; ^3^National R&D Branch Center for Pelagic Aquatic Products Processing (Hangzhou), Hangzhou, China; ^4^Institute of Sericultural and Tea, Zhejiang Academy of Agricultural Sciences, Hangzhou, China; ^5^Department of Food Science and Technology, National University of Singapore, Singapore, Singapore

**Keywords:** shellfish polysaccharides, extraction and purification, lipid metabolism, metabolic disorders, shellfish

## Abstract

Shellfish are diverse, widely distributed organisms that are a rich source of biological resources. Polysaccharides are an important components in shellfish, hence a great deal of attention has been directed at isolation and characterization of shellfish polysaccharides because of their numerous health benefits. Differences in shellfish species, habits, and environment result in the diversity of the structure and composition of polysaccharides. Thus, shellfish polysaccharides possess special biological activities. Studies have shown that shellfish polysaccharides exert biological activities, including antioxidant, antitumor, immune-regulation, hypolipidemic, antihypertensive, and antihyperglycemic effects, and are widely used in cosmetics, health products, and medicine. This review spotlights the extraction and purification methods of shellfish polysaccharides and analyses their structures, biological activities and conformational relationships; discusses the regulatory mechanism of shellfish polysaccharides on hyperlipidemia, hypertension, and hyperglycemia caused by lipid metabolism disorders; and summarizes its alleviation of lipid metabolism-related diseases. This review provides a reference for the in-depth development and utilization of shellfish polysaccharides as a functional food to regulate lipid metabolism-related diseases. To achieve high value utilization of marine shellfish resources while actively promoting the development of marine biological industry and health industry.

## Introduction

Shellfish belong to the phylum Mollusca, which comprises about 110,000 species of shellfish worldwide, including crustaceans and mollusks (1). Shellfish have been extensively cultured and are widely distributed worldwide. They can be classified under seawater, freshwater, and terrestrial shellfish, based on the environment where they are found. Seawater shellfish are the predominant type among all shellfish. According to the China Fishery Statistical Yearbook 2020 (2), seawater shellfish aquaculture accounts for approximately 70% of the mariculture production, which is an important component. At present, there are about 30 species of known marine shellfish, and those cultivated on a large scale include mussels, oysters, scallops, clams, razor clams, and abalone. China is one of the leaders in the breeding and processing of marine shellfish. Its abundant shellfish resources provide a good raw material base for the development of bioactive substances from shellfish.

Proteins, lipids, polysaccharides, and nucleic acids are the four basic components of life, which play a crucial role in anti-aging, and as antivirals (3) and antioxidants (4). Shellfish is rich in bioactive substances, including taurine, polysaccharides, polypeptides, and fatty acids among others (5). Different species, habits, and the living environment led to the complex composition and structure of shellfish polysaccharides, which contribute to the unique diversity in the biological activity and functional characteristics of shellfish polysaccharides (6). Of late, several studies have been focusing on shellfish polysaccharides in China. The results indicate that shellfish polysaccharides have antibacterial, anti-aging, antioxidant, immunomodulatory, hypolipidemic, antihypertensive, and anti-obesity effects that can reduce liver damage and exert other pharmacological effects (7).

Currently, there has been an increased attention to metabolic syndrome (MetS), which is a cluster of metabolic disorders associated with obesity, insulin resistance, hypertension, hyperlipemia, hyperglycemia, type II diabetes, and cardiovascular disease (8). MetS are metabolic disorders of a complicated pathological nature that results from an imbalance in proteins, fats, carbohydrates, and other substances imbalance. The prevalence of MetS is steadily increasing because of an increased prevalence of obesity and insulin resistance. The characteristics of MetS include: (i) the common pathological cause of MetS is obesity, particularly central obesity, which can leads to the development of insulin resistance and hyperinsulinemia. (ii) The key factors contributing to the risk of MetS are vascular dysfunction, atherosclerotic dyslipidemia, elevated plasma glucose levels, prethrombotic state, and vascular inflammation (9). At present, the development of naturally occurring compounds for the treatment of MetS is a research hotspot, especially in the fields of medicine and functional foods. Polysaccharides, especially those isolated from marine organisms, are reported to be non-toxic and harmless and are useful in the management of MetS (10). The Function of shellfish polysaccharides to improve MetS are shown in Table 1.

**TABLE 1:** Shellfish polysaccharides improve the functional activity of Mets.

Sources	Polysaccharide type	Metabolic syndrome	Medical uses	Functional activity	References
*Bellamya quadrata*	Sulfated polysaccharide	Atherosclerotic plaque	Stabilize atherosclerotic plaque	Enhance the stability of atherosclerotic plaque by promoting autophagy	(13)
*Crassostrea gigas*	Glucose polymer	Hypertension	Antihypertensive	Decrease systolic and diastolic blood pressure	(84)
		Hypoglycemic	Anti-diabetic	Regulates carbohydrate metabolism improves insulin sensitivity, reduces insulin resistance, and improves fasting blood glucose levels	(109)
*Patinopecten yessoensis*	Acidic polysaccharide	Hyperlipemia	Prevent obesity	Regulation of the gut microbiota promotes the production of SCFAs and improves lipid metabolism in the serum and amino acid metabolism in the cecum.	(124)
*Crassostrea gigas*	–	Alcoholic steatohepatitis	Protecting liver function	Restores the gut barrier, decreased proinflammation levels, and reduced lipid accumulation by activating the AMPKα/SREBP1c pathway and enriching SCFAs.	(45)
Abalone and Scallops	Glycosaminoglycans	Obesity Diabetes	Anti-obesity, improvement glucose tolerance, and anti-cerebrovascular disease	Improves the ecology of the intestinal flora and promotes the multiplication of beneficial bacteria. Regulates the expression of fat-forming related factors to inhibit fat formation.	(125)
Oysters	Chitosan		Prevention of obesity, diabetes and other metabolic disorders	Fermentation of the intestinal microbiota to increase the concentration of SCFAs, thereby regulating hormone secretion and altering the structure of the gut microbiota.	(74)

Research progress related to the separation, purification, structural analysis, and biological activity of shellfish polysaccharides are expounded in this review. Different extraction methods for the isolation of shellfish polysaccharides based on their structural composition, molecular weight, and functional groups have been compared in this review. Herein, we have summarized how shellfish polysaccharides can alleviate lipid metabolism disorders via multiple mechanisms such as regulating the expression of relevant genes, inhibiting the activity of specific enzymes, and regulating protein channels and reducing lipid oxidation. We have also elaborated on the role of shellfish polysaccharides in reducing the levels of lipids, blood pressure, and plasma glucose. The aim of this review is to provide a reference for the further development and utilization of shellfish polysaccharides as functional foods in the management of lipid metabolism-related diseases.

## Extraction and purification of shellfish polysaccharides

The commonly used methods for the extraction of shellfish polysaccharides include aqueous extraction, ethanol-subsiding method, ultrasonic-assisted enzymatic extraction, enzymatic hydrolysis, and alkali extraction. Shellfish polysaccharides obtained from different types of shellfish are significantly different with respect to their structural composition, functional groups, linkages, and molecular weights (11). Okamoto et al. (12) conducted a comprehensive glycosaminoglycan analysis using 10 shellfish, namely *Ruditapes philippinarum*, *Scapharca broughtonii*, *Turbo cornutus*, *Crassostrea nippona*, *Corbicula japonica*, *Mizuhopecten yessoensis*, *Mytilus galloprovinciali*s, *Crassostrea gigas* (oyster), *Neptunea intersculpta*, and *Pseudocardium sachalinense.* Their findings show that the composition and content of polysaccharides vary among shellfish species. Furthermore, the protease enzyme assisted extraction was employed to obtain the *Bellamya quadrata* polysaccharides (BQPS). BQPS possessed a total polysaccharide content of 91.88 ± 1.23%, sulfuric acid group of 9.12 ± 1.59%, 91.1 kDa. BQPS was only consisted of glucose without any protein (13). *Mactra veneriformis* polysaccharides (MVPS) was extracted by the method of water decoction and alcohol precipitation, polysaccharide contents of the fractions were over 99.8%. The average molecular weight values of the purified polysaccharides MVPS-1, MVPS-2, and MVPS-3 were estimated to be 446, 426, and 452 kDa, respectively (14). Thus, the extraction methods are strongly related to the structural composition and biological activity of the polysaccharides present in shellfish.

### Aqueous extraction

Aqueous extraction involves the use of hot or cold water as a solvent to extract. Aqueous extraction is associated with benefits such as low cost, simple equipment, and no reagent residue. However, the extraction time is long and hydrolysis of the peptide chain in the polysaccharides is challenging. Moreover, the extraction yields are significantly lower compared with other methods. Aqueous extraction is suitable for extracting samples having high sugar content. Getachew et al. (15) used subcritical water (SW) to extract a bioactive polysaccharide from *C. gigas*, which has an α-(1→4) configuration of D-glucan, consisting of only one monosaccharide unit and having an average molar mass of 1.08 × 10^5^ Da. The response surface method (RSM) was used to optimize the extraction process. The predicted optimal extraction conditions were 125.01°C, 14.93 min, and solid:liquid ratio of 44.69:1 (mL/g). Using the above method, the predicted yield of the polysaccharide from *C. gigas* was 18.66%. The structural composition of polysaccharides may be varied with different extraction methods.

Previous studies have shown that the polysaccharide components BYL-S and BYL-J were extracted from *Neverita didyma* using aqueous extraction at 60°C and alkaline extraction at 25°C with 1% Na_2_CO_3_. Four polysaccharide components (BYL-S1, BYL-S2, BYL-S3, BYL-S4) were obtained by subjecting the extract to anion exchange column chromatography using a DEAE Sepharose FF anion-exchange column without glyoxylates and sulfate groups. Glc was the only monosaccharide that made up the isolated polysaccharides. BYL-S1 and BYL-S2 with relative molecular weights of 721 and 642 kDa, which were obtained by aqueous extraction, respectively. Analysis of the chemical structure of BYL-S2 revealed that BYL-S2 had α-(1→4) as the main chain and showed the presence of a few β-(1→3,4)- and β-(1→3)-branched D-glucans of the pyran type. However, the monosaccharide composition of BYL-J was relatively complex, containing Man, GlcN, GalN, Gal, and Fuc in addition to Glc. The molar ratio Glc:Man:GlcN:GalN:Gal:Fuc was 78.9:1.7:3.4:2.2:5.2:5.6 (16).

Similarly, Gao et al. (17) used water at 25°C and at 60°C, as well as dilute alkali to extract *C. gigas* polysaccharides and obtained yields of 16.1, 1.9, and 13.3%, respectively. All three crude polysaccharides were glucans with molecular weights of 2.1 × 10^6^ Da, 2.0 × 10^6^ Da, and 9.8 × 10^5^ Da. Subsequently, five purified polysaccharide fractions, namely CT-11, CT-21, RT-11, RT-21, and JT-11, were obtained using DEAE Sepharose FF anion exchange chromatography and Sepharcryl S-400 gel chromatography. CT-11 was mainly composed of →4) -α-D-GLC - (1→ and →3, 4) -β-D-GLC (1→ and →2, 4) -β-D-GLC (1→ pyranoglucan. A crude polysaccharide was extracted from the clam *Hyriopsis cumingii* Lea using aqueous extraction. Then, the water-soluble purified polysaccharide HCLPS-1 was obtained by separation and sequential purification using DEAE-cellulose and Sephadex-G200 gel-permeation chromatography. HCLPS-1 was found to be a homogeneous polysaccharide with an average molecular weight of 1.56 × 10^5^ Da, with a (1→4)-linked β-D-glucopyranosyl residues in the main chain. By acetylating the monosaccharides for GC, HCLPS-1 was determined to be consisted of D-glucose and D-xylose in a 35:1 molar ratio (18).

### Ethanol-subsiding method

The ethanol-subsiding method involves the addition of different concentrations of ethanol into a concentrated aqueous extract to reduce the solubility of shellfish polysaccharides in the alcoholic solution, followed by precipitation and refining the polysaccharides after solid-liquid separation. Mussel polysaccharide (MP-A) is an α-D-pyranic glucan. The main chain consists of an α-(1→4) glycosidic bond and each of the 12 monosaccharides connects D-glucose to this chain through an α-(1→2) glycosidic bond, forming a branch. It has been found that the molecular weight of MP-A is about 1.2 × 10^3^ kDa (19). MP-A is not very stable in aqueous solution and the sugar chains can easily hydrolyze to yield low-molecular-weight polysaccharides and even oligosaccharides and monosaccharides. Liu et al. (20) optimized the extraction of the mussel polysaccharide using the ethanol-subsiding method. The results showed that the highest yield was 12.52% of the dry mass of mussel when extracted at 100°C using a 1:6 ratio of material: liquid and a single extraction lasting 3 h. Wang et al. (21) used the ethanol-subsiding method combined with trypsin enzymolysis to extract *C. gigas* polysaccharides from internal organs (CGOP). Their results showed that the total sugar content of CGOP was 43.92% when the water:material ratio was 3:1, the amount of enzyme was 0.8%, and when extracted in a water bath for 5 h. Another research group used 95% ethanol and performed an overnight extraction at 4°C to extract and precipitate the polysaccharides from the mucilage of the mud snail Bullacta exarata. DEAE-cellulose ion exchange and size-exclusion chromatography were used to further purify the extracted polysaccharides. Three polysaccharides, namely BEMPB, BEMPB1, and BEMPB2 were obtained. BEMPA was a mannose-containing polysaccharide with several (1→3, 4)-linkages, having a molecular weight of 22,977 Da. BEMPB1 has a molecular weight of 64,117 Da and was determined to be an arabinose-containing polysaccharide with several (1→3)-linkages. BEMPB2 was primarily consisted of (1→3, 4)-linked mannoses and was determined to have a molecular weight of 47,507 Da (22).

### Enzymatic hydrolysis

Enzymatic hydrolysis mainly uses the specific selection function of proteases to hydrolyze the corresponding chemical bond of the peptide, reducing the resistance from the cell wall, cell membrane, and the intercellular matrix during solvent extraction. Enzymatic hydrolysis is a relatively simple process to obtain high yields of the active components as traditional extraction methods are generally unsuitable in the hydrolysis of shellfish polysaccharides and in effectively breaking the peptide chains that are connected with the polysaccharide backbone (23, 24). RSM was used to optimize the method used to extract polysaccharides from *Mytilus edulis* (mussel). A liquid ratio of 9.8 mL/g, enzymolysis temperature of 46°C, enzymolysis time of 133 min, and pH of 5.9 resulted in a yield of 19.2%, which was not significantly different from the theoretical values obtained using the simulation equation (25). Another research group developed an enzyme-assisted process to extract *Sinonovacula constricta* (razor clam) polysaccharides (SCPs) using RSM. SCPs were obtained at a yield of 17.72% using the following optimized parameters for enzymatic digestion: 173.0 min, pH 8.2, 50.0°C, enzyme content 4.0%. The crude polysaccharide was purified by DEAE-52 column to obtain the neutral polysaccharide SCP-1 and the acidic polysaccharide SCP-2, which was composed of glucose, sulfate, and uronic acid. SCP-1 and SCP-2 have relative molecular weights of 248.7 and 275.6 kDa, respectively. The differences between enzyme-assisted extraction and aqueous extraction were compared using UV-visible and IR spectroscopy. Interesting, the yield from enzymatic extraction was 12.26% higher than that of aqueous extraction (26). Enzymatic extraction can maintain the integrity of SCP and improve the yield of the polysaccharide as compared to traditional extraction methods.

Some previous studies have also reported the extraction of crude polysaccharides from *Haliotis Discus* (abalone) *Hannai Ino* gonad using a combination of protease hydrolysis and boiling-methanol extraction. The extracted polysaccharides were purified to homogeneity using gel-filtration chromatography on Sephacryl-S 100 and Sephacryl-S 200 columns, and using semipreparative reverse-phase high-performance liquid chromatography (semi-RP-HPLC) equipped with a SOURCE 30Q anion exchange column. The average molecular weight of sulfated polysaccharide (PAGP) was 12.5 kDa. The carbohydrate component of PAGP was consisted of rhamnose (Rha), fucose (Fuc), and galactose (Gal) in a 1.0:1.6:2.3 molar ratio (27). Thus, an improved method combining the enzyme hydrolysis and boiling-methanol extraction methods was used to obtain crude polysaccharides from *Haliotis Discus Hannai* Ino gonad. The crude polysaccharides extracted by this method contained 6.1% of protein, whereas those extracted using protease-hydrolysis, water extraction, and ethanol precipitation were found to contain 18.6% of protein (28).

### Ultrasonic-assisted enzymatic extraction

Ultrasonic-assisted enzymatic extraction (UAEE) is an innovative method that couples ultrasonic waves and enzyme extraction by enzymatic hydrolysis and ultrasonic vibration cavitation to extract polysaccharides (29, 30). Moreover, this method can be used to effectively extract polysaccharides without destroying the biological activity of molecules (31). UAEE was used to isolate the polysaccharides from *Mytilus coruscus* (thick-shell mussel). Subsequently, the optimum conditions for the extraction of *M. coruscus* polysaccharides (MCPs) were determined using RSM experiments. The ultrasonic power was 60 W, 36°min, 64°C, liquid: material ratio was 30 mL/g, enzyme concentration was 3.2%, which led to a polysaccharide yield of 12.86% ± 0.12%. A new polysaccharide (MCP1-2) was isolated after purification on an AB-8 macroporous resin, DEAE Sepharose Fast Flow, and Sepharose CL-6B column. The molecular weight of MCP1-2 was 134.9 kDa, and the polysaccharide was found to be composed of mannose, rhamnose, glucuronic acid, glucose, galactose, and L-Fuc in a 1.53:1:4.83:81.82:2.36:1.51 molar ratio. Furthermore, infrared (IR) and NMR spectroscopy confirmed the presence of α- and β-configurations in MCP1-2. The molecular weight of MCP1-2 was found to be one-tenth that of MP-1 extracted using hot water.

Ultrasonic treatment led to polysaccharide degradation and a subsequent decrease in molecular weight (32). Enzyme-assisted extraction (EAE) and UAEE were used to extract crude polysaccharides from *Corbicula fluminea* (Asian Clam). The results showed that the molecular weights of polysaccharides EP and EP-us were 500–620 kDa and 343–473 kDa, which contained different proportions of mannose, glucose, galactose, fucose, proteins, and sulfate groups, respectively (33). It was found that EP extracted using EAE had a higher molecular weight, lower sulfate content and lower antioxidant activity compared to EP-us extracted using UAEE. This was probably due to the degradation of EP-us and additional changes to its chemical structure resulting from ultrasound irradiation (34). The extraction temperature was 62°C, 32 min, ultrasound power was set to 300 W, and the water: raw material ratio was 35 mL/g, which afforded a polysaccharide yield of 36.8% using UAEE. Compared with EAE, UAEE is more effective in extracting crude polysaccharides and can be used to obtain higher polysaccharide yields in a shorter extraction time.

### Alkaline extraction method

The alkaline extraction method mainly hydrolyzes the ester bonds of the sugar protein, which facilitates the separation of polysaccharides in solution. However, excessive alkalinity can damage the polysaccharide structure and reduce extraction yields. Water, alkali, and enzymes have often been used to extract the polysaccharides of *Crassostrea rivularis* (oyster). Previous studies have found that the KOH extraction method has the highest extraction efficiency, suggesting that alkali treatment can break down the protein molecules linked to glycogen, and promote the breaking of the N- and O-connected bonds. The optimal extraction conditions for this procedure were as follows: 30% KOH (w/v), 90°C, and 120 min. The polysaccharide yield was 92.36 ± 0.13%. Subsequently, the crude polysaccharides were purified using column chromatography using DEAE-52 cellulose and Sepharose 2B gel. Separated OG1, OG2, and OG3 with average molecular weights of 1,660, 2,270, and 2,330 kDa, respectively. It was found that OG1 and OG3 were mainly composed of rhamnose, but OG2 showed the presence of glycogen with α−(1→4) −D-linked glucose. Branching of the polysaccharide chain was seen in each 6.5 glucose residues on average, which is →4, 6) −α−D−Glc−(1→ and a trace amount in α-D-Glc−(1→ branched units (35). However, polysaccharides underwent enolization, and C4 and C5 underwent dehydration when the KOH concentration exceeded 30%. Unsaturated sugar alcohol groups were subsequently formed, leading to an increase in extract viscosity and making it difficult to filter, resulting in low yields. Previous studies have shown that divalent cations (Ca^2+^, Mg^2+^) have a stronger effect on the aggregation of sulfated polysaccharides than monovalent cations (Na^+^, K^+^). Therefore, cations with low-valence bonds should be selected for alkaline extraction to prevent denaturation of sulfate polysaccharides (36).

Variations in shellfish species and the living environment lead to the formation of polysaccharides with different structures, molecular weights, and biological activities. Some studies have shown that the content, yield, extraction, and purification of shellfish polysaccharides are not only affected by the properties of the raw materials, but are also related to shellfish site, procurement time, storage, and pretreatment conditions. Therefore, an extraction method should be chosen carefully to maximize purification efficiency and final yields. The difference is shown in Table 2.

**TABLE 2:** Comparison of the methods used to extract shellfish polysaccharides.

Shellfish species	Extraction and purification methods	Advantages	Disadvantages	Extraction yields	Extraction purification efficiency	References
Oyster sinensis, Mactra veneriformis (Reeue)	Aqueous extraction	Low cost, simple equipment, and no reagent residue	Long extraction time and incomplete separation	Low	Low	(14, 17)
*Bullacta exarate*, *Crassostrea gigas*, *Ostrea rivularis* Gould	Ethanol-subsiding method	Economical, simple, and easy to operate	Some of the small polysaccharides are removed	Low	Low	(22, 126, 127)
Sinonovacula constricta, Chinese clam	Enzymatic hydrolysis	The effective ingredients are released completely and can remove the protein linked to polysaccharides	High costs and time consuming	High	High	(26, 128)
*Corbicula fluminea*, Razor clams	Ultrasonic-assisted enzymatic extraction	Rapid extraction of polysaccharides	Generation of low-molecular-weight polysaccharides	High	High	(33, 129)
Chinese clam, Mytilus edulis	Alkaline extraction method	Rapid hydrolysis of polypeptides, leading to the release of polysaccharides	Excessive alkalinity disrupts the molecular structure	High	High	(130, 131)

## Biological activity of shellfish polysaccharides

Shellfish contain abundant levels of polysaccharides, and most of these compounds can be isolated by combining extraction, separation, and purification techniques. Shellfish polysaccharide activity is not only related to the presence of sulfate functional groups, but also to the structural attributes, molecular weight, substitution pattern, branching, and monosaccharide composition. Therefore, the biological activities of shellfish polysaccharides are diverse and include antioxidant, antitumor, antiviral, antiaging, hypoglycemic, hypolipidemic, and hypotensive functions (37). The structures and biological activities of shellfish polysaccharides are shown in Table 3.

**TABLE 3:** Structural analysis and biological activities of shellfish polysaccharides.

Species	Source	Extraction method	Polysaccharide type	Polysaccharide backbone structure	Molecular Weight (kDa)	Monosaccharide	Biological activity	References
*Haliotis discus hannai* Ino (Pacific abalone)	Abalone gonad	–	Uronic acid-containing polysaccharide	→4)-β-GlcA(1→2)-α-Man(1→ repeating unit with Fuc, Xyl, and Gal in the branch.	–	Man, Gal, GlcA, Xyl, Fuc and Rha	Antioxidant, antitumor, anticoagulant	(132)
*Haliotis discus Hannai Ino*	Abalone viscera	Protease-assisted aqueous extraction	Sulfated heteroglycan	1,3-linked rhamnose and 1,3,6-linked galactose	11	Glucose, fucose, xylose, rhamnose and galactose with molar ratio of 1.0:2.0:3.9:6.7:7.4	Antioxidant, appetite-suppressive	(133)
*Corbicula fluminea*	Whole species	Enzymatic hydrolysis	Acidic proteoglycan	Mainly containing 14.2% aspartic acid, 16.3% glutamic acid, 14.4% cysteine, and 9.0% valine.	61.5–2113.4	Glc, GlcN and Man with a molar ratio of 10.8:4.4:1.0	Protect the liver from hepatocyte injury induced by alcohol	(134)
*Meretrix meretrix* (Linnaeus)	soft tissues, including mantle, foot, gill, adductor, and viscera	hot water extraction	Glycosaminoglycans	α(1→4)–Glc, α(1→4,6)-branched Glc, and terminal Man or Gal residues	272–257	Glc and Gal in a 50:1→M ratio	immune-enhancing activity	(126)
*Meretrix meretrix Linnaeus* (Veneridae)	Whole species after removing the shells	Enzymatic extraction	Homogenous polysaccharide	(1→4)-linked-α-D-glucopyranosyl residues	510	D-glucose and D-galactose residues at a molar ratio of 3.51:1.00	Immunological activity	(135)
*Mactra veneriformis*	Flesh materials	Water extraction and alcohol precipitation	Glucosan analogs	[→4Glc1 →4Glc1 →4Glc1 → 2Glc1→ 4Glc1→4Glc1 →4Glc1] n	446, 426, 450	D-galactose	Immune-enhancing, anti-hyperglycemic	(136)
*Mytilus coruscus* (thick-shell mussel)	Mussel meat	Ultrasonic-assisted enzymatic extraction	–	Mannose, rhamnose, glucuronic acid, glucose, galactose, and L-fucose	134.9	Mannose, rhamnose, glucuronic acid, glucose, galactose, and L-Fuc at a molar ratio of 1.53:1:4.83:81.82:2.36:1.51	Antioxidant, reduction in lipid accumulation in the liver	(32)
*Mytilus coruscus*	Mussel meat	Hot-water extraction	α-(1→4)-D-glucan	(1→4)-α-D-Glc	1350	D-glucose	antioxidant activity	(137)
*Mytilus coruscus*	Mussel meat	Ethanol-subsiding method	Homopolysaccharide – glucan	(1→4)-α-D-glucose	1200	α-D-glucan	Prevention of NAFLD	(43)
*Perna viridis*	Whole viscera	Enzymatic extraction	Acidic polysaccharides with sulfate groups	–	689	Glucosamine, uronic acid, and galactosamine with molar ratio of 3.7:2.6:1	Hypolipidemic and antioxidative activity	(56)
*Bullacta exarata*	Flesh	Enzymatic extraction	Sulfated polysaccharides	–	BEP1:21.7, BEP2:46.1, BEP3: 62.2	BEP1: Rha, Arb, Man, Glu, Fuc and Gal in the mole ratio of 36: 34: 14: 6: 5: 5. BEP2: Man, Arb, Fuc and Gal (47, 22, 16, and 15 mol%, respectively). BEP3: of Rha, Man, Arb, Glu, and Fuc in the mole ratio of 35: 39: 11: 8: 7	Antitumor and antioxidant activities	(138)
*Crassostrea gigas*	*Crassostrea gigas* muscle	Subcritical water extraction	D-glucan with α-(1→→→4) configuration	–	10.8	Glucose	Antihypertensive, and hypoglycemic activities	(15)
*Crassostrea gigas*	Whole species after removing the shells	Hot water extraction	Uniform glucose polymer	α-configuration of D-glucan	6500	Glucose	Improved hepatic injury	(139)
*Crassostrea gigas*	Whole species after removing the shells	Aqueous two-phase system	–	(1→4)-α-D-glucosyl backbone and branching points located at *O*-3 of glucose with a terminal-D-Glcp.	3.48	Glucose	Anti-tumor activities	(140)
*Crassostrea gigas*	Oyster meat, consisting of the adductor muscle and mantle	Enzymatic extraction	–	→4)-α-D-Glc-(1→ with few →3,4)-β-D-Glc-(1→and→2,4)-β-D-Glc-(1→branched units)	3413	Glucose	Antihypertensive effect;	(84)
Pacific oyster *Crassostrea gigas* (Thunberg)	Whole species after removing the shells	Hot-water extraction	β-glucans	–	435	β-glucose	Food allergies.	(141)
*Bellamya quadrata*	Whole species after removing the shells	Enzymatic assisted extraction	Sulfated polysaccharide	–	91.1	Glucose	Prevention of atherosclerotic plaques	(13)
*Cipangopaludina chinensis*	Flesh	Hot-water extraction	Sulfated polysaccharide	(1→3)-linked α-D-Glc with sulfate radical content of 9.12%.	91.1	Glucose	Antiangiogenic effects	(142)
*Corbicula fluminea*	Whole species after removing the shells	Ultrasonic-assisted enzymatic extraction	Sulfated fucans	–	EP: 500-620 EP-us: 343-473	Fucose, arabinose, mannose, glucose, and galactose with molar ratios of 1.34:0.63:3.53:45.20:4.84(EP) and 2.42:1.68:4.85:39.08:6.99(EP-us).	Superoxide radical-scavenging activity	(33)
*Ostrea rivularis* Gould	Whole species after removing the shells	Enzyme-assisted extraction	–	–	118	Glucose (76.3%) and galactose (23.7%).	Antioxidant activities	(128)
*Meretrix casta* (Chemnitz)	Whole species after removing the shells	Ethanol-subsiding method	–	Maltodextrins, maltose, maltotriose, maltotetraose, maltopentose, and maltohexose	–	–	Improves gut flora	(143)
*Bellamya purificata*	Foot muscle	Ethanol-subsiding method	–	BPS-1: α-(1→4)-linked glucose residues, with branches at C-6 consisting of terminal and α-(1→3)-linked glucose residues. BPS-2: α-(1→4)-linked glucose residues, with branches at C-3 and C-6, consisting of terminal and α-(1→3)-linked glucose residues.	BS-1: 7200 BS-2: 8300	BPS-1 was composed of glucose, fucose, arabiinose and xylose with a molar ratio of 99:2:1:1. BPS-2 was composed of glucose, fucose, arabinose, galactose, xylsoe and mannose with a molar ratio of 218:5:3:2:1:1	Anti-inflammatory effects	(144)
*mussel*	Mussel meat	Enzymatic extraction and Ethanol-subsiding method	Glucosan analogs	The glycosidic bond of →4)-α-D-Glcp-(1→, and the end group α-D-Glcp-(1→ and α-D-Glcp-(1→6) -α-D-Glcp-(1→pass→4,6)-α-D-Glcp-(1→. The O-6 bond is connected to the main chain.	4.25	Glucose (0.995), galactose (0.003), glucosamine hydrochloride (0.001), and galactose hydrochloride (0.001) glucose (0.995), galactose (0.003), glucosamine hydrochloride (0.001), and galactose hydrochloride (0.001) glucose (0.995), galactose (0.003), glucosamine hydrochloride (0.001), and galactose hydrochloride (0.001) glucose (0.995), galactose (0.003), glucosamine hydrochloride (0.001), and galactose hydrochloride (0.001)	Immunomodulatory activity	(57, 145)
*Patinopecten yessoensis*	*Patinopecten yessoensis* skirt	Hot water extraction and enzymatic extraction	Acidic polysaccharide	→ 4)β-GlcA(1 → 4)α-GlcNAc → and → 4)β-GlcA(1 → 3)β-GalNAc →	13.58	The molar ratio of Man, GlcN, Rha, GlcA, GalN, Glc, Gal, and Fuc was 17.7: 11.7: 12.1: 1.0: 15.9: 8.0: 23.7: 24.7	Prevents obesity	(124)

### Hypolipidemic effect of shellfish polysaccharides

Hyperlipidemia is characterized by abnormal lipid metabolism or transport, resulting in elevated levels of one or more lipids in the serum (38). Excessive consumption of a high-fat diet decreases the levels of the antioxidant enzymes, leading to increased oxidative stress and dyslipidemia. Hyperlipidemia is caused by an imbalance between the production of reactive oxygen species (ROS) and lipoprotein metabolism, and is clinically characterized by abnormally high plasma levels of triacylglycerols (TGs), cholesterol (TC), free fatty acids (FFA), and low-density lipoprotein cholesterol (LDL-C), along with an increased accumulation of TG in hepatocytes (39). At the same time, hyperlipidemia can trigger fatty liver disease, atherosclerosis, coronary heart disease, and other cardiovascular and cerebrovascular diseases, seriously endangering human health. Therefore, regulating lipid metabolism is particularly important for the prevention or treatment of hyperlipidemia. Previous studies have found that shellfish polysaccharides can exert hypolipidemic effects, thereby preventing hyperlipidemia and related disorders arising from MetS.

#### Regulation of lipase activity

Lipid metabolism in the body is regulated by several important enzymes, and the extent of enzyme activity affects the TG, TC, and fatty acid levels in the body. Lipoprotein lipase (LPL) and hepatic lipase (HL) degrade triglycerides; hydroxymethylglutaryl CoA reductase (HMGCR) and acetyl CoA carboxylase (ACC) inhibit hepatic cholesterol and fatty acid synthesis, respectively (40). The sterol regulatory element-binding protein (SREBP) in the endoplasmic reticulum is a conditionally activated transcription factor and has three subtypes, namely, SREBP-1a, SREBP-1c, and SREBP-2. Of these, the main regulators are SREBP-1a and SREBP-2, which control the expression of genes, including 3-hydroxy-3-methylglutaryl-CoA reductase (HMGCR) and low-density lipoprotein receptor (LDLR), which are involved in cholesterol synthesis. In addition, SREBP-1c can bind to the promoters of several genes regulating the lipogenic enzymes and induce their expression (41), thereby regulating fatty acid synthesis, and TG and glucose metabolism. SREBP-1c has emerged as an important factor involved in the insulin regulation of lipogenic enzyme expression (42).

**FIGURE 1:**
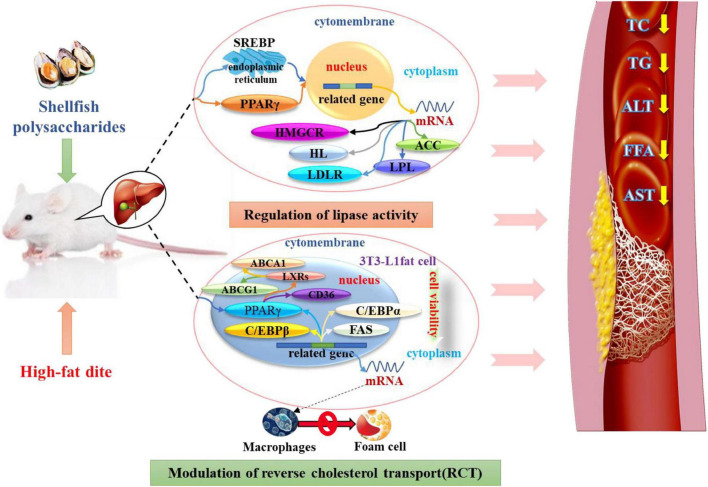
Shellfish polysaccharides regulate lipase and RCT lipid-lowering pathways.

Wu et al. (43) explored the effect of α-D-glucan (MP-A) extracted from the thick-shelled mussel Mytilus on non-alcoholic fatty liver disease (NAFLD). MP-A consists of (1→4)-α-D-glucose as the main chain, and a D-glucose unit was linked to this chain by α-1, 2-glucosidic bonds after every twelve monosaccharides to form a branch. The results demonstrated that MP-A significantly remodeled the structure of intestinal flora at different taxonomic levels, enhanced microbial diversity, and altered the composition of the intestinal flora, effectively alleviating NAFLD by maintaining homeostasis of the intestinal flora and regulating the relevant gut-liver axis signaling pathways. Moreover, MP-A can promote the production of short-chain fatty acids (SCFAs) in the intestine, which in turn regulates lipid metabolism-related protein expression (44). After transport to the liver, it can inhibit PPARγ and SREBP-1c expression and activity, regulate lipid metabolism disorders by inducing SREBP-1c transcription factor, alleviate excessive hepatic lipid deposition, and regulate intrahepatic lipid metabolism (43). Similarly, oyster polysaccharides were found to help restore the intestinal barrier, reduce the levels of the pro-inflammatory markers, and decrease lipid accumulation by activating the AMPKα/SREBP1c pathway and enriching SCFAs (45). A study has reported that the aqueous extract of the blue mussel, Mytilus edulis, shows anti-adipogenic activity and inhibits lipid accumulation by suppressing the expression of the mRNA levels of the adipogenic genes (46). This may be likely due to the induction of SREBP-1c transcription factor by shellfish polysaccharides, which regulate adipocyte LPL mRNA expression, thus leading to corresponding changes in the rate of LPL synthesis and its activity such that the TG levels decrease (Figure 1).

#### Modulation of reverse cholesterol transport

Reverse cholesterol transport (RCT) occurs in the macrophages, which can “swallow” lipids (containing large amounts of cholesterol) in the blood vessels, become foam cells, and adhere to the arterial vessel wall, which is a prominent feature of atherosclerosis (AS). The main pathway of cholesterol metabolism is through binding to the ATP binding cassette transporter A1 (ABCA1), a cholesterol transport carrier on the cell membrane, transporting to the exogenous cholesterol acceptors and transferring to HDL. This may prompt the excretion of cholesterol from the body, thereby reducing the formation of AS plaques. In cells, cholesterol efflux is mainly mediated through the peroxisome proliferator-activated receptor γ (PPARγ)-LXR α-ABCA1/ABCG1 pathway, and PPARγ-LXR α-ABCA1 plays an important role in modulating RCT (47). The nuclear transcription factor PPAR γ is highly expressed in both macrophages and foam cells. It regulates multiple cholesterol metabolism genes in macrophages, including a scavenger receptor for cholesterol absorption genes, such as CD36, and cholesterol efflux genes including ABCA1 (48).

A previous study has reported that the aqueous extract of Mytilus edulis (BME) inhibited 3T3-L1 adipogenesis by regulating the Wnt/β-catenin signaling pathway and suppressing PPRAγ levels. The aqueous extract also reduced viability and decreased the accumulation of lipids, TGs, and total cholesterol in 3T3-L1 cells (46). Furthermore, the extract significantly suppressed the mRNA expression levels of lipogenic genes including C/EBPβ, PPARγ, C/EBPα, and FAS in 3T3-L1 mature adipocytes. BME could also inhibit lipid accumulation in 3T3-L1 adipocytes after differentiation, particularly in the early and midstages (49). In addition, as oysters are rich in chitosan, clams can reduce the mRNA expression levels of PPARγ and LXRα in white adipose tissue. By inhibiting high-fat diet-induced adipocyte differentiation in obese rats, BME was found to improve dyslipidemia and prevent weight gain (50). This effect may have been mediated by reducing the viability of 3T3-L1 cells, regulating the mRNA expression of PPARγ, preventing the transformation of macrophages into foam cells, and preventing cholesterol deposition in the arterial wall, which drives cholesterol out of the body (Figure 1).

#### Reduction of lipid peroxidation

Lipid peroxidation is a process in which excess free radicals in living organisms combine with unsaturated fatty acids in the presence of various stimuli to constantly generate lipid peroxides such as malondialdehyde (MDA). Superoxide dismutase (SOD) is an active protein that can naturally trap and scavenge oxygen free radicals and protect cells from damage. Glutathione peroxidase (GSH-Px) scavenges H_2_O_2_ and reduces OH- radical production (51). Shellfish polysaccharides can promote SOD release from the cell surface and scavenge ROS, thereby preventing free radical-initiated chain reactions and inhibiting lipid peroxidation. The crude polysaccharides from Cyclina sinensis (CSPS) have been obtained by using the following central composite design. The crude CSPS was then sequentially purified by chromatography using DEAE-52 and Sephadex G-100, which afforded three purified fractions, namely, CSPS-1, CSPS-2, and CSPS-3. CSPS-3 showed the highest superoxide and hydroxyl radical-scavenging activity *in vitro* compared with the other two polysaccharides. The activity of CSPS-3 may be likely attributed to its relatively high protein, uronic acid, sulfate, and complicated monosaccharide composition, which led to its structural and functional properties being completely different from CSPS-1 and CSPS-2 (52).

In an *in vivo* study in tilapia, the hexbengal polysaccharide was found to increase serum SOD and GSH-Px activities, decrease MDA levels, and increase the ability of SOD and GSH to scavenge hydroxyl radicals and superoxide radicals, thereby highlighting its antioxidant effects. In addition, the DPPH radical scavenging of hydroxyl radicals and superoxide anion (O2–), the anti-lipid peroxidation effect in liver tissue, and the total antioxidant capacity of the extract were found to gradually increase with an increase in polysaccharide concentration (53). In a previous study, Ruditapes philippiarum fractions were centrifuged to obtain three components: cytoplasm, nucleus, and mitochondria. These centrifuged components were separated and purified using a combination of enzymatic hydrolysis and DEAE-Sepharose Fast Flow anion exchange column chromatography. The authors report that the polysaccharides in the cytoplasm and nucleus had little difference in their monosaccharide composition. The main monosaccharides were glucosamine, galacturonic acid, and galactosamine. The composition of the mitochondrial polysaccharide was relatively simple and was found to be composed of only glucose. Moreover, at a concentration of 2–12 mg/mL, the *in vitro* antioxidant activity of the three-component polysaccharide showed an upward trend as the polysaccharide concentration was increased. However, there were differences in the antioxidant activity of the three polysaccharides likely due to differences in the composition, molecular weight, degree of polymerization, and main chain characteristics (54).

The high-fat diet enhanced lipid peroxidation and protein damage (carbonyl) in the cerebral cortex and hippocampus of mice, reduced the non-enzymatic antioxidant defenses (sulfhydryl) in the cerebral cortex and cerebellum, reduced CAT and SOD activities in the entire brain tissue, and enhanced nitric oxide (NO) production in the cerebral tissues (55). It was found that the SOD, GSH-Px, and CAT activities in brain tissue were increased by 11, 21, and 32%, respectively, after the administration of 200 mg/kg of Perna viridis (Asian green mussel) polysaccharides (PVPs) to mice fed a cholesterol-supplemented diet. These findings suggested that PVP could suppress the high cholesterol diet-induced increase in thiobarbituric acid reactive substance (TBARS) by blocking lipid peroxidation through activation of the antioxidant enzymes (56). MDA can inhibit SOD and GSH-Px activities, weaken their antioxidant effects, and aggravate the damage caused by oxygen free radicals. Furthermore, our results found that mussel polysaccharide promoted the mRNA expression of GSH-Px, SOD, Nrf2 and HO-1, increased CAT, GSH-Px, SOD levels and decreased MDA levels (57). Nrf2 is a transcription factor that regulates redox homeostasis, which induces repair and degrades damaged macromolecules (58). SOD, CAT, and GSH-Px are key antioxidants in the fight against oxidative stress, scavenging free radicals, converting ROSs into non-toxic compounds and protecting the body from oxidative damage. Therefore, the mechanism of reducing lipid oxidation by shellfish polysaccharides may be attributed to the induction of Nrf2 signaling pathway, enhancement of SOD and GSH-Px activities, elimination of the metabolites generated by free radicals, reduction in MDA levels, alleviation of the inhibitory effect of MDA on SOD and GSH-Px activity, thereby decreasing blood lipid levels.

#### Reduction of cholesterol and triglyceride levels, and regulation of lipoprotein levels

TC, TG, low-density lipoproteins (LDLs), and high-density lipoproteins (HDLs) affect the body fat content. A decrease in serum HDL levels, and an increase in TC, TG, and LDL levels are important factors inducing atherosclerosis and cardiovascular and cerebrovascular diseases. The link between dyslipidemia and atherosclerosis is well established, as elevated LDL stimulates endothelial cells to secrete atherogenic cytokines, which promote the formation of foam cells in the intima, the first step in the development of atherosclerotic lesions. HDL-C can activate lecithin cholesterol acyltransferase, catalyze the interaction of the β-fatty acyl group in lecithin with cholesterol to generate cholesterol lipids, reduce serum cholesterol and triglyceride levels, and transport cholesterols from the surrounding tissues, and even atherosclerotic plaque, to the liver for circulation or excretion in the form of cholic acid through RCT. LCAT is an enzyme that catalyzes the esterification of cholesterol with unsaturated fatty acids. It plays an important role in HDL-C metabolism and the reverse transport of TC. Moreover, LCAT expression determines the production of HDL-C and affects the elimination of TC. When LCAT expression decreases, lipid metabolism in the blood is disordered, which leads to the accumulation of TC and acceleration of the rate of lipid formation (59). Li et al. (60) administered low (50 mg/kg BW⋅d), medium (100 mg/kg BW⋅d), and high (200 mg/kg BW⋅d) doses of glycosaminoglycan from Perna viridis to high-fat diet-fed mice. The results showed that TC, TG, LDL-C levels in serum, and TC and TG levels in the livers of mice in the high-dose group decreased. Serum HDL-C and LCAT activities increased. Over an appropriate range, it was found that the higher the concentration of polysaccharide, the better was the curative effect, indicating the effective regulation of lipid metabolism in the livers of hypercholesterolemic mice (60).

In a previous study, glycosaminoglycan from Patinopecten yessoensis was administered to hyperlipidemic mice at low (100 mg/kg⋅d), medium (200 mg/kg⋅d), and high (400 mg/kg⋅d) doses. The results showed that the medium and high doses of glycosaminoglycan could significantly reduce serum TC, TG, and LDL-C levels in hyperlipidemic mice. Among them, mice that were administered a high dose of scallop glycosaminoglycans showed a reduction in serum TC, TG, and LDL-C levels by 35.38, 31.07, and 22.58% respectively, and an increase in HDL-C level by 41.18%. Thus, the glycosaminoglycan of P. yessoensis was effective in reducing blood lipid levels in hyperlipidemic mice (61). Therefore, the shellfish polysaccharide could reduce hyperlipidemia by increasing LCAT activity, promoting serum HDL-C production, reducing serum and liver TC and TG levels, and maintaining lipid levels in the body.

#### Appropriate activation of autophagy

Autophagy is a compensatory self-protection and life-sustaining catabolic process that maintains cell homeostasis. Autophagy disorders are closely related to a variety of disease pathologies, such as tumors, type 2 diabetes, and obesity (62). Recently, autophagy has been considered to be an important mechanism of atherosclerotic plaque stabilization (63). The formation and development of atherosclerotic plaques result from the gradual accumulation of abnormal lipids within the arterial walls (64). Moderate autophagy activation can effectively stabilize atherosclerotic plaques by regulating macrophage inhibition of the inflammatory response. P62 is an adaptor protein and autophagy-related marker, which can bind to autophagosome and be phagocytized during the lysosomal-degradation pathway after binding to ubiquitin and LC3 (65). Therefore, the downregulation of LC3B and upregulation of P62 expression are considered indicators of enhanced autophagy flux.

Interestingly, compared with non-sulfated polysaccharides, sulfated polysaccharides have lower flexibility, greater polarity, and higher electronegativity due to the presence of sulfate groups. These unique physical and chemical properties confer specific biological activities related to the prevention and treatment of AS (66). The authors in this study found that the long-term exposure to high salt and hypoxic conditions enriched shellfish meat with sulfated polysaccharides, which imparted unique anti-inflammatory, lipid-lowering, and antithrombotic effects. The crude polysaccharide from Bellamya quadrata (Cipangopaludina chinensis, CBQP) was extracted by protease-assisted extraction; the ratio of water:raw material was 24:1, enzyme dosage was 285 U/g, and enzymolysis was conducted at pH 4.7 at 67°C. CBQP was further purified to obtain the target polysaccharide BQPS using Q Sepharose Fast Flow and Sephacryl S-400 gel column chromatography. The sulfate content was determined to be 9.12 ± 1.59% and the molecular weight was 91.1 kDa (13), which was attributed to the sulfuric acid component and the 1→3-glycoside bond (67). The above results suggested that BQPS could inhibit LC3B expression and increase P62 expression in a dose-dependent manner, and also enhance the autophagy flux. Moreover, BQPS yield of up to 1.42% was obtained with the sulfuric acid group content as high as 9.12 ± 1.59%. The enhancement of BQPS-induced autophagy in atherosclerotic plaques was found to be selective with appropriate intensity. The stability of atherosclerotic plaques was found to be greatly enhanced by promoting autophagy. More importantly, BQPS was found to significantly decrease AI, shrink atherosclerotic plaques, and reduce VI value.

#### Regulation of fatty acids and intestinal flora

**FIGURE 2:**
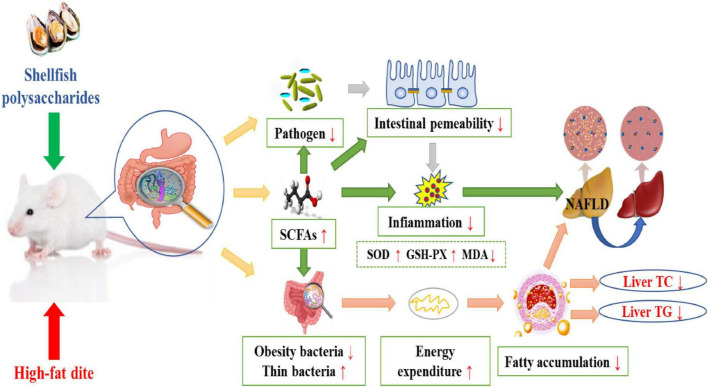
Mechanism of shellfish polysaccharides in regulating the intestinal microflora and lowering blood lipids.

The gut is the largest reservoir of microorganisms in a host. Thousands of different microbial species in the gut play a crucial role in providing nutrition, directing innate immunity, and regulating metabolism (68, 69). Dysbiosis of the intestinal flora may trigger immune system diseases through the action of T cells in the gut mucosa (69). Studies have shown that alterations in the composition of gut flora are associated with inflammatory diseases including obesity and diabetes (70). Additionally, the intestinal flora is associated with the regulation of fat storage, which is associated with hyperlipidemia, obesity, and insulin resistance (71, 72). Therefore, regulating the composition of the intestinal flora can effectively reduce the lipid levels in the body. The regulation pathway is shown in Figure 2. Oyster polysaccharides extracted from raw and steamed oysters (RPS and SPS) were found to modulate the intestinal flora, SCFAs, and intestinal barrier in a mouse model of alcoholic liver injury. Restructuring of the gut microbiome and an elevation in SCFAs levels, which are essential for the prevention of alcohol-induced steatohepatitis, were found in the RPS- and SPS-treated groups. The oyster polysaccharides could also help restore the intestinal barrier, reduce inflammation, and decrease lipid accumulation by activating the AMPKα/SREBP1c pathway and enriching SCFAs (45). After polysaccharide treatment, the TG levels in liver tissues were found to decrease by 17.88 and 15.69% in the RPS and SPS groups, respectively. SCFAs have been shown to modulate hepatic triglyceride levels by mediating the expression of the genes and proteins of lipogenic enzymes (73). Moreover, SCFAs can alter the intestinal microflora by bringing about changes in the existing percentage of specific microorganisms (74). Collectively, these results demonstrated that oyster polysaccharides could modulate the gut-liver-metabolic axis to inhibit alcoholic hepatic steatosis and decrease fat content.

Hyperlipidemia refers to abnormal lipid metabolism caused by aberrant lipid metabolism or transport, which increases the levels of one or more lipids in the serum. The mechanisms of shellfish polysaccharides in regulating hyperlipidemia mainly include: (i) Induction of SREBP transcription factor in the endoplasmic network to regulate enzyme activity related to fat metabolism, resulting in the inhibition of fat formation and reduction in fat content; (ii) reduction in the activity of 3T3-L1 cells, regulation of the mRNA expression of PPAR and RCT, prevention of macrophages from transforming into foam cells, inhibition of cholesterol deposition on the arterial wall, and promotion of cholesterol excretion *in vitro*; (iii) enhancement of SOD and GSH-Px activities, elimination of the metabolites of free radicals and oxygen free radicals in the body, reduction in MDA and blood lipid levels; (iv) improvement in LCAT activity, promoting serum HDL-C production, and decrease in TC and TG levels in serum and liver; (v) regulation of fatty acids and intestinal flora, promotion of the secretion of intestinal endocrine hormones, reduction in plasma glucose levels and lipid metabolism, and inhibition of weight gain. Therefore, shellfish polysaccharides can be used as naturally occurring hypolipidemic agents to regulate lipid levels and maintain normal body weight (Figure 2).

### Antihypertensive effects of shellfish polysaccharides

Hypertension is a serious cardiovascular disorder characterized by elevated systemic arterial pressure, which can lead to many chronic conditions, such as heart disease and stroke, with a long incubation period. Hypertension results from the repeated elevation in blood pressure exceeding 140/90 mm Hg (75). In hypertensive patients, the efficacy of coronary vasodilators is reduced and the minimum coronary resistance is increased. Changes in coronary vascular tone or endothelial dysfunction are factors affecting coronary vasodilation (76). The major biochemical factors leading to hypertension include increased sympathetic nervous system activity, inadequate dietary intake of calcium and potassium, altered renin secretion leading to elevated renin-angiotensin system (RAS) activity, increased angiotensin-converting enzyme (ACE) activity resulting in the overproduction of angiotensin II (Ang II) and deactivation of the kallikrein-kinin system (KKS), endothelial dysfunction and deficiencies of vasodilators including reduced NO bioavailability, abnormalities in vessel resistance due to vascular inflammation, increased activity of vascular growth factors, and altered cellular ion channel (77). High blood pressure can be controlled by the use of antihypertensive drugs (78); however, these drugs are associated with side effects, such as headaches, tachycardia, dizziness, cough, taste disorders, and rashes (79). It has been reported that shellfish polysaccharides are potent ACE inhibitors and can regulate NO levels; thus, these compounds have the potential to be developed as functional foods to prevent hypertension.

#### Inhibition of angiotensin-converting enzyme

**FIGURE 3:**
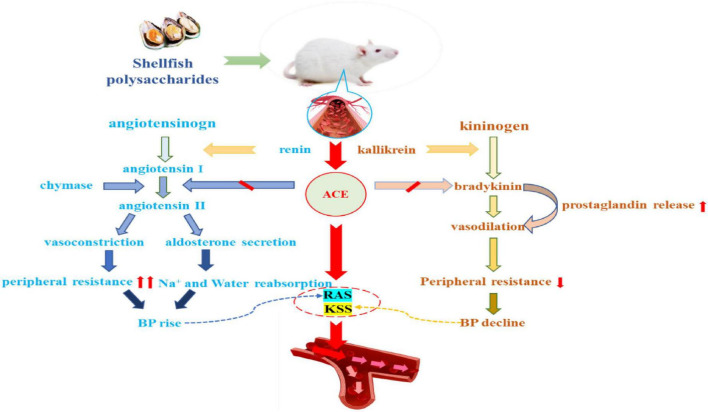
Shellfish polysaccharides inhibit angiotensin-converting enzyme and regulate blood pressure.

Recent studies have shown that hypertension can be treated by inhibiting ACE (80). ACE is a key regulator of the RAS, which plays a key role in the transformation of angiotensin I (Ang I) to Ang II (81). Moreover, ACE can convert the decapeptide (Ang I) into octapeptide (Ang II) via the RAS and KKS. More importantly, Ang II is a vasoconstrictor peptide that causes high blood pressure and plays a key role in the occurrence of myocardial dysfunction and cardiac arrest (82). ACE can also inactivate bradykinin, which is a vasodilator in the KKS (83). Shellfish polysaccharides can inhibit ACE and prevent the conversion of Ang I to Ang II, which is an effective method to reduce hypertension. As shown in Figure 3.

Recent studies have reported *C. gigas* polysaccharide to be a homogeneous glucose polymer with a molecular weight of approximately 3.413 × 10^6^ Da, which can regulate systolic and diastolic blood pressure and exert an antihypertensive effect. Thus, this polysaccharide can potentially be used as a therapeutic agent for the management of hypertension (84). Similarly, the polysaccharide from oyster obtained by cooking, acid hydrolysis, alcohol precipitation, and drying, has a molecular weight of 3.93–10.84 × 10^6^ Da and is mainly connected by α-(1→4) glycosidic bonds and has a branch of α-(1→6) glycoside bond. This molecule can regulate systolic and diastolic pressure and reduce hypertension (85). Ji et al. found that the compound powder of oyster polysaccharide from Andrias davidianus containing the active peptide had a synergistic effect in inhibiting ACE, indicating the antihypertensive effect of the oyster (86). Moreover, Ma et al. (87) used high-performance liquid chromatography (HPLC) to quantify hippuric acid produced by the reaction of ACE with the tripeptide substrate Hip-His-Leu (HHL) to determine the inhibitory activity of the enzyme. The results showed that ACE inhibition increased gradually in a concentration-dependent manner with the increase in oyster polysaccharide concentration, resulting in the effective lowering of blood pressure.

Previous studies have reported that shellfish are rich in chitosan, which is a polymer obtained from the partial deacetylation of *N*-acetylglucosamine prepared by the basic deacetylation of chitin. Among the reported compounds, chitosan oligosaccharides (COS), a derivative of chitosan (mainly composed of glucosamine units of polycation polymer), is synthesized by the chemical or enzymatic hydrolysis of chitosan. Studies on chitosan and its derivatives have identified their potential as ACE inhibitors. Hong et al. (88) studied the ACE-inhibitory activity of different COS and identified that chitosan trimer is more effective in lowering blood pressure compared to other oligomers. In addition to ACE, renin plays a vital role in the RAS. Renin (angiotensinogenase) is a rate-limiting enzyme in the RAS. It cleaves plasma angiotensinogen to Ang I, which is further converted to Ang II by ACE. Therefore, renin inhibition is also an attractive target in hypertension therapy (79). Studies show that 90% deacetylated and medium molecular weight (1,000–5,000 Da) COS exhibit the strongest renin inhibition (IC50 = 0.51 mg/mL) and act as a competitive inhibitor of the enzyme (89). Collectively, it can be concluded that COS derivatives can be developed as functional foods for the effective management of hypertension.

#### Nitric oxide in regulating vasodilation

Endothelial dysfunction directly leads to MetS and complications, including cardiovascular disease, insulin resistance, and hypertension. NO synthases catalyze the production of NO from L-arginine, and is a key regulator of vasodilation. Impairment in endothelial function may be caused by the reduced production/release of endothelium-dependent relaxing factors (NO and prostacyclin PGI2) and the excessive production of endothelial-dependent contractile factors (including ROS) (90). Previous studies have shown that increasing the production of vasodilator factors (NO and prostaglandins) can regulate endothelial function and reduce the production of vasoconstrictor factors (ET-1) to relieve blood pressure (91). Upon release by the endothelium, NO can activate guanylate cyclase and increase cyclic guanosine monophosphate in vascular smooth muscle cells, leading to the activation of several types of K + channels, including calcium-activated potassium channels, ATP-dependent K + channels (KATP), and voltage-dependent K + channels (KV) (92). Opening of K + channels hyperpolarize the smooth muscle cell membranes, which closes the voltage-dependent Ca2 + channels and relaxes smooth muscle cells (93).

**FIGURE 4:**
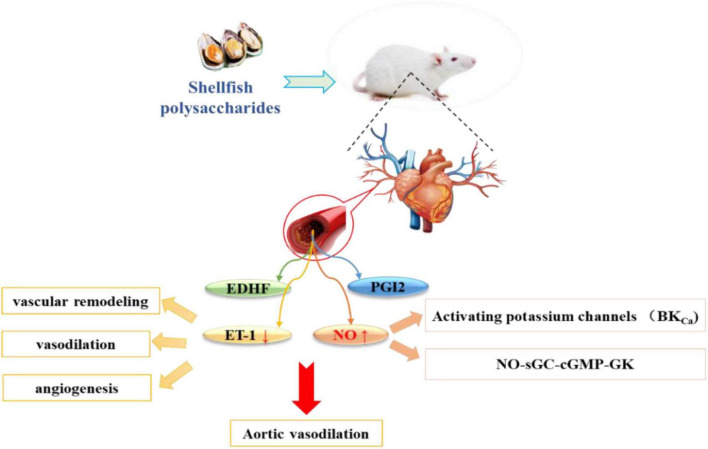
Shellfish polysaccharides regulate vasodilation factors and reduce high blood pressure.

In addition, NO induces relaxation of the vascular smooth muscles through the activation of sGC, thereby converting GTP to cGMP, which acts as a second messenger and activates PKG. The latter, in turn, phosphorylates various proteins and produces vasodilation. The NO-sGC-cGMP-PGK pathway and/or membrane hyperpolarization via K + channel activation was found to induce rat aortic vasodilation (94). The effects of Scapharca subcrenata (AS) and Tegillarca granosa (GA) on high-fat diet-induced endothelial dysfunction were studied. The results showed that AS and GA could increase the expression level of vasodilation-related proteins and inhibit the expression of ET-1 protein to enhance vasoactivity and improve endothelial dysfunction, thereby reducing hypertension (95). Currently, only a few studies have reported the hypertensive effects of shellfish polysaccharides through the regulation of NO (Figure 4); thus, the detailed mechanism is pending further investigation.

Thus, shellfish polysaccharides can effectively reduce hypertension and be used to prevent or treat this condition. The main approaches include: (i) prevention of the conversion of Ang I to Ang II by inhibiting ACE; regulation of systolic and diastolic blood pressure *in vivo*, leading to a decrease in hypertension. (ii) regulation of the vasodilator factor NO that affects the contraction and tension of vascular smooth muscle cells of blood vessels, thereby reducing hypertension. The current research exploring the mechanism of shellfish polysaccharides in reducing hypertension is not adequate; thus, further studies are needed to provide a more definite basis for the use of shellfish polysaccharides as functional foods.

### Hypoglycemic effects of shellfish polysaccharides

Diabetes is a chronic disorder caused by defects in carbohydrate, lipid, and protein metabolism. It can be classified into type I and type II diabetes. Type I diabetes is characterized by insufficient insulin production or the inability of the pancreas to produce insulin. The insulin secretion in type II diabetes is normal, but cells and tissues are resistant to insulin, which results in a spike in blood glucose levels. Strategies in the management of diabetes include the inhibition of α-amylase, α-glucosidase, glucosidase, glycogen synthase kinase 3β (GSK-3β), lipase, aldose reductase, protein tyrosine phosphatases 1B (PTP1B), and dipeptidyl peptidase IV (DPP-4). Moreover, the prevention of insulin resistance, increase in the phosphorylation of AMP-activated protein kinase (AMPK), regulation of glucose and lipid metabolism, reduction in oxidative stress, and protection of β-pancreatic cells are other approaches to control hyperglycemia (96). In recent years, researchers have considered the bioactive compounds from shellfish polysaccharides in the development of new functional foods.

#### Reduction of adipocytokines in plasma

Adipose tissue is an important endocrine organ that secretes multiple adipocytokines that affect energy metabolism and insulin sensitivity (97, 98). Hypertrophic adipocytes increase plasma leptin, TNF-α, and IL-6 levels, but decrease plasma adiponectin levels (99). Hormone-sensitive lipase (HSL) is an enzyme that promotes the degradation of adipose tissue and lipolysis of triglycerides to monoacylglycerol and free fatty acids. HSL activity in type II diabetes is low, which may be a mechanism of obesity in accelerating diabetes (100).

A high-fructose diet may lead to lipolysis and lipid metabolism disorder, reduce the lipolysis rate of visceral adipocytes, increase the accumulation of TG in adipocytes, and promote the enlargement of adipocytes (101). Studies have shown that raw and steamed oyster polysaccharides (RPS and SPS) were administered via oral gavage at a dose 282 mg/kg.bw after 28 days in a mouse model of alcoholic liver injury. The results showed that alcohol suppressed the levels of propionic and butyric acids in the serum and feces of mice, but no changes were observed in the levels of acetate, isobutyric acid, valeric acid, and isovaleric acid. Treatment with oyster polysaccharides significantly increased propionate levels and enhanced the production of propionic and butyric acid microorganisms (*Lactobacillus reuteri*, *Bifidobacterium longum*, and *Roseburia* spp.) (45). Butyrate affected the expression of genes involved in the regulation of lipid/glucose metabolism (102). RPS and SPS treatment increased butyrate levels, increased adipose tissue lipolysis, reduced adipose tissue weight, reversed the increase in TNF-α and IL-6 and the decrease in adiponectin levels in hyperlipidemic rats.

Moreover, it has been reported that changes in hormone levels might activate the PPARs/PI3K/AKT signaling pathway, inhibit the expression of the downstream signal CD36, and promote GLUT4 expression. These effects have been reported to enhance the sensitivity of the liver to insulin, leading to active glucose and lipid metabolism (103). More importantly, shellfish polysaccharides could prevent diet-induced diabetes through the gut microflora-mediated signaling pathways and by a reduction in lipopolysaccharide levels in diabetic rats by reducing the abundance of *Proteobacteria* in the gut. These studies suggested that shellfish polysaccharides could prevent the development of diabetes by decreasing plasma adipocytokines and decreasing the abundance of intestinal flora.

#### Reduction in lipid peroxidation

The abnormal expression of inflammatory factors and oxygen free radicals can cause macrophage infiltration in adipose tissue, block the insulin signaling pathway, lead to insulin resistance, and induce type II diabetes (104). Studies have found that ROS production and antioxidant defense ability in diabetic patients are affected, leading to an increase in oxidative stress index levels. Therefore, antioxidants play an important role in alleviating the symptoms of diabetes (105).

Previous studies have reported the ultrasonic-assisted extraction of the visceral crude polysaccharides of Abalone (AVP) and intragastric administration of crude AVP to diabetic mice to study its hypoglycemic activity. It was found that AVP could significantly reduce the blood glucose levels in diabetic mice and improve abnormal glucose tolerance of mice over a certain range. Furthermore, AVP administration was found to improve SOD and GSH-Px activities and enhance the free radical scavenging effects and the inhibition of lipid peroxidation in diabetic mice. Moreover, with an increase in AVP dose, the antioxidant capacity was found to increase. Thus, AVP may reduce damage to the islet β cells in the pancreas by scavenging oxygen free radicals and inhibiting lipid oxidation, thereby reducing blood glucose levels in diabetic mice (106). Dong et al. (107) administered low (100 mg/kg⋅d), medium (200 mg/kg⋅d), and high (400 mg/kg⋅d) doses of the glucosaminoglycan from Paphia undulate (PUG-1) by oral gavage to D-galactose-induced aging mice. It was found that PUG-1 was effective in scavenging hydroxyl radicals, superoxide anion radicals, and DPPH radicals *in vitro*, with an IC50 of 4.23, 3.19, and 4.36 mg/mL, respectively. Furthermore, it is important to note that compared with the aging model group, PUG-1 significantly increased the SOD, GSH-Px, and CAT levels in the serum, livers, and brains of aging mice (107). The results of these studies suggest that shellfish polysaccharides can reduce blood glucose levels in hyperglycemic mice, and that the mechanism of hypoglycemia may be by protecting the islet β cells by enhancing free-radical scavenging ability and reducing oxidative stress. PUG-1 can also promote glucose utilization in peripheral tissues, improve insulin sensitivity and increase the number of insulin receptors, and improve insulin resistance.

#### Inhibition of digestive enzymes

α-Amylase and α-glucosidase are digestive enzymes. Carbohydrates, such as starch, are first degraded into smaller oligosaccharides by α-amylase, and then into monosaccharides by α-glucosidase, which leads to a spike in blood glucose levels. Therefore, inhibiting the digestive enzymes is a promising therapy for diabetes (108). Accordingly, the hypoglycemic effect of shellfish polysaccharides can be evaluated based on their inhibitory effect on α-amylase and α-glucosidase. *C. gigas* polysaccharides (CGPs) extracted using subcritical water has been reported to have antidiabetic activity. An increase in the concentration of CGPs from 0.625 to 10 mg/mL led to an increase in enzyme inhibition from 26.51 to 90.53% and from 16.21 to 78.68% for α-amylase and α-glucosidase, respectively. CGP administration led to a higher inhibition of α-amylase (IC50 = 1.58 ± 0.03mg/mL) compared with α-glucosidase inhibition (IC50 = 2.77 ± 0.01mg/mL) (15). In addition, the hypoglycemic activity of CGPs was also confirmed using metabolomics analysis of diabetic mice (109). Therefore, CGP shows potential as a naturally occurring hypoglycemic agent. However, the efficacy and mechanism of CGP having different molecular weights need to be further studied. Onchidium struma polysaccharide (OSP) was extracted using ultrasonic-assisted extraction. The monosaccharides of OSP consisted of mannose, rhamnose, glucuronic acid, galacturonic acid, glucose, and fucose with an average molecular weight of 1.34 × 106 Da. The inhibitory activity of OSP against α-glucosidase has shown to be positively correlated with its concentration; at 40 and 50 μg/mL, the rate of inhibition was 72.8% ± 5.0% and 82.2% ± 3.3%, respectively, which makes it a promising candidate in its use as a functional food as well as in drug development (110). Few studies have reported the inhibitory effects of OSP with different molecular weights on digestive enzymes. Polysaccharide-enzyme complexes were formed mainly by hydrophobic interactions and hydrogen bonding spontaneously (111). Tremella fuciformis polysaccharides have been found to alter the secondary structure of α-amylase, making it more rigid, less flexible and more compact. The enzyme activity was inhibited by affecting the formation of the active center (112). However, the structure of the shellfish polysaccharide and specific details regarding its mechanism of enzyme inhibition are unclear and need further study.

#### Regulation of amino acid and fatty acid metabolism

Amino acid metabolism results in the synthesis of unique proteins, peptides, and other nitrogen-containing substances in the body through deamination, transamination, combined deamination, decarboxylation, or degradation into α-keto acids, amines, and carbon dioxide. Furthermore, the metabolites can be transformed into sugars or lipids, or resynthesized into non-essential amino acids. Fat metabolism is the process of digestion, absorption, synthesis, and degradation of fat in the body by various related enzymes, which are then processed into substances required by the body to ensure normal physiological function. Diabetes mellitus is a metabolic disorder characterized by disturbances in the metabolism of carbohydrates, lipids, and proteins. Therefore, abnormal amino acid and fat metabolism may cause an increase in plasma glucose levels and result in diabetes.

Elevation in amino acid metabolite levels leads to the production of 3-hydroxyanthranilic acid, 2-hydroxyphenylacetic acid, propionylglycine, and 3-hydroxyanthranilic acid as intermediate products of l-tryptophan catabolism and are excreted in the urine. 2-Hydroxyphenylacetic acid is related to tyrosine metabolism. Besides, acylglycines, such as propionylglycine and phenylacetylglycine, have been identified as potential biomarkers in disorders of amino acid and fatty acid metabolism. It is generally believed that glycine or carnitine can combine with excessive intermediates generated from amino acid or fatty acid metabolism to promote excretion or balance coenzyme A. Thence, acylglycines in urine were considered biomarkers signifying the accumulation of the relevant acyl CoA esters (113). CGPs are homogeneous glucose polymers with an average molecular weight of 6.5 × 106 Da, and comprising primarily of →4)-α-D-Glc-(1→, with a few →3,4)-β-D-Glc-(1→ and →2,4)-β-D-Glc (1→ branched units (114). Based on the metabolite changes, the mechanism of action of the polysaccharide could be determined (115). Using metabonomics, Zhao et al. (109) explored the dynamic changes of endogenous small molecule metabolites in urine. Diabetic mice were treated with low (200 mg/kg), medium (400 mg/kg), and high (800 mg/kg) doses of CGPs. Their findings suggested that the model group had excessive excretion of allantoic acid/alantoic acid, which was manifested as the effect of hyperglycemia on purine metabolism. Compared with the model group, ornithine, methylguanidine, and l-pipecolic acid levels in the high-dose CGP group were found to be increased, likely due to the treatment of this group with high-dose CGP, which resulted in recovery and reduction in urine volume.

Pantothenate is an anion B-group vitamin that is important for the synthesis of coenzyme A and the metabolism of proteins, carbohydrates, and fats. A study has reported that diabetes increases the risk of atherosclerosis and that pantothenate levels in the urine of mice with atherosclerosis were significantly decreased through suppression of the TCA cycle (116). It was found that pantothenate levels in urine were increased in the group treated with a high dose of CGPs, indicating that its efficacy in the management of diabetes by inhibiting the development of atherosclerosis. Moreover, previous studies have shown that the L-carnitine can partially improve the metabolic indices in diabetes. Treatment with L-carnitine was found to completely normalize plasma cholesterol, triglyceride, free carnitine, and TBARS levels, but only partially restored blood glucose levels in diabetic rats (117). The results indicated that CGP treatment significantly increased L-carnitine levels in mice and played a role in the treatment of diabetes. Therefore, shellfish polysaccharides can alleviate diabetes-induced polyuria, inhibit atherosclerosis development, and improve diabetes by regulating amino acid and fatty acid metabolism. However, further studies are warranted to understand the efficacy and mechanism of action of shellfish polysaccharides with different molecular weights.

#### Regulation of insulin resistance

Under physiological conditions, insulin regulates glucose homeostasis by enhancing glucose uptake in insulin-sensitive tissues, and also regulates nutrient delivery by vasodilation in small feeding arteries. Specifically, insulin-mediated production of NO from the endothelium leads to increased blood flow and enhanced glucose disposal. In general, insulin resistance is considered to be a decrease in the sensitivity or responsiveness to insulin metabolism including insulin-mediated glucose disposal. Typically, insulin resistance is considered as a decrease in sensitivity or responsiveness to the metabolic actions of insulin including insulin-mediated glucose disposal (118). Insulin resistance can affect glucose and lipid metabolism. The sensitivity of the surrounding tissues to insulin decreases, which leads to abnormal glucose metabolism. At the same time, the abnormal output of liver glucose leads to increased blood glucose levels and is accompanied by dyslipidemia, leading to diabetes and MetS (119).

Previous studies have reported that an increase in adipose tissue is associated with increased oxidative stress and the secretion of pro-inflammatory cytokines, such as interleukin (IL)-6 and tumor necrosis factor-alpha (TNF-α), and causes direct and indirect insulin resistance via inhibiting insulin signaling (120). Another study supports the findings that the high-fat diet of obese rats can cause an imbalance of adipocytokines and significantly increase circulating leptin levels, thereby leading to metabolic disorders related to insulin resistance (121). Adipose tissue is an important endocrine organ that secretes many cytokines including adipokines and hormones. In obese individuals, the fat cells in adipose tissue become abnormal and exhibit hypertrophy, thus storing a large quantity of lipids. At the same time, adipose tissues are infiltrated by a large number of macrophages. The macrophages in adipose tissue play a pro-inflammatory role and cause metabolic disorders. The increase in the adipose tissue mass can induce the infiltration of macrophages and secretion of pro-inflammatory cytokines in obese individuals, thereby promoting ROS generation (122), which also induces insulin resistance in adipose tissues and skeletal muscle, leading to impaired insulin secretion and atherosclerosis (123).

In addition, compared with non-inflammatory macrophages, the pro-inflammatory macrophages were found to secrete more exosomes and induce insulin resistance in mature mouse embryonic fibroblasts (3T3-L1), causing impaired insulin secretion and atherosclerosis. Shellfish polysaccharides possibly reduce oxidative stress and inflammation by promoting antioxidant enzyme activity, decreasing the expression of adipogenic transcription factors including PPARγ and C/EBPα in 3T3-L1 pre-adipocytes, reversing the increase in serum TNF-α and IL-6 levels, and enhancing insulin sensitivity and LPL activity to prevent obesity-related insulin resistance, which leads to increased glucose uptake in muscle and adipose tissue and the lowering of serum glucose levels. However, the mechanism of how shellfish polysaccharides regulate macrophages and enhance insulin sensitivity is still unclear and needs to be further studied.

**FIGURE 5:**
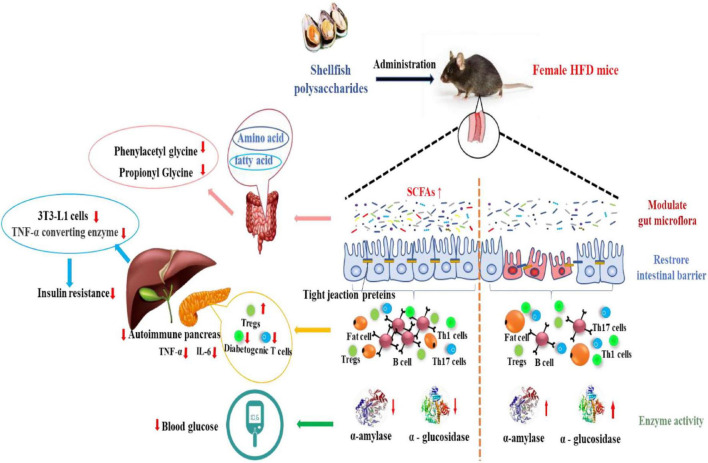
Shellfish polysaccharides reduce hyperglycemia pathways.

To summarize, as shown in Figure 5, shellfish polysaccharides reduce hyperglycemia mainly through: (i) improving lipolysis rate in adipose tissue and reducing adipose tissue weight, thereby regulating plasma adipocytokines and improving glucose and lipid metabolism; (ii) enhancing free radical scavenging activity and protecting pancreatic islet β cells; (iii) inhibiting the activity of the digestive enzymes and reducing high blood glucose levels; (iv) improving metabolic function by regulating amino acid and fatty acid metabolism; (v) reducing insulin resistance and enhancing tissue sensitivity to insulin. The diversity of shellfish polysaccharide activity and special physiological functions can effectively and significantly reduce plasma glucose levels, providing a basis for the research and development of functional foods in the management of type II diabetes. The structural heterogeneity of shellfish polysaccharides and their regulation of sugar metabolism have gradually become research hot spots globally.

## Conclusion and future perspectives

Different species, habitats, and living environments of shellfish lead to differences in the composition, structure, and molecular weight of shellfish polysaccharides, conferring upon them unique and diverse biological activity and functional characteristics. In recent decades, with increasing research on the development of marine biological resources, the consumption and demand for seafood shows an upward trend worldwide. Compared with terrestrial organisms, the living environment of marine organisms is more complicated, which makes marine resources exhibit unique characteristics and biological activities, leading to the discovery of more significant advantages. Currently, several studies exist delineating the positive effects of shellfish polysaccharides in alleviating disorders in lipid metabolism; however, the specific mechanisms of their hypolipidemic, antihypertensive, and hypoglycemic need to be further studied. Besides, the structure, biological activity, and mechanism of action of shellfish polysaccharides from different shellfish have not been fully elucidated, and their structural modifications have not been well explored. Moreover, an understanding of the role of shellfish polysaccharides in lipid metabolism disorders is based on *in vitro* or *in vivo* data, and additional human clinical studies are needed to accurately verify their efficacy. Studies on the functional properties of the active components of shellfish polysaccharides will help further promote their development as functional foods, increase the utilization of shellfish substances, and further expand the use of active substances from marine organisms. These functional properties can be used by food scientists to develop new foods to reduce the increased prevalence of diet-related diseases in rapidly developing economies such as China and India. In the future, the active components from shellfish polysaccharides will likely make significant contributions to human health. This review can provide a reference for the further development and utilization of shellfish polysaccharides as functional foods to regulate lipid metabolism.

## Author contributions

XX and QJ consulted the literature and drafted the manuscript. HY, XZ, LC, YC, and HC revised the article. SL conceptualized the idea, drafted the outline, and revised the manuscript. All authors contributed to the article and approved the submitted version.
